# Armodafinil as a Potential Pharmacological Treatment for Attention Deficit Hyperactivity Disorder in Adults: A Review

**DOI:** 10.2174/1570159X22666240131121642

**Published:** 2024-03-14

**Authors:** Reyna Lamas-Aguilar, Araceli Diaz-Ruiz, Luz Navarro, Raúl Miranda-Ojeda, María de los Ángeles Martínez-Cárdenas, Alfonso Mata-Bermudez, Camilo Rios

**Affiliations:** 1 Departamento de Neuroquímica, Instituto Nacional de Neurología y Neurocirugía Manuel Velasco Suárez, Ciudad de México, México;; 2 Departamento de Fisiología, Facultad de Medicina, Universidad Nacional Autónoma de México,Ciudad de México, México;; 3 The Mind Project, Office for Equity, Diversity, Inclusion, and Belonging, Harvard University, Smith Campus Center, Cambridge, Massachusetts, USA;; 4 Faculty of Medicine, Autonomous University of Mexico State, Toluca de Lerdo, Estado de Mexico, Mexico;; 5 Departamento de Atención a la Salud, Universidad Autónoma Metropolitana Unidad Xochimilco, Ciudad de México, México;; 6 Dirección de Investigación, Instituto Nacional de Rehabilitación Luis Guillermo Ibarra Ibarra., Ciudad de México, México

**Keywords:** Attention deficit hyperactivity disorder, circadian rhythms, sleep problems, cognitive disturbances, psychostimulant drugs, armodafinil

## Abstract

**Introduction:**

Armodafinil is a psychostimulant that promotes alertness, and it has been shown to improve attention, memory, and fatigue in healthy adults and adults with neurodevelopmental conditions that share symptoms with Attention Deficit Hyperactivity Disorder (ADHD). It is generally well tolerated and safe, and most of the adverse events reported are considered not serious. However, the available evidence on the efficacy of armodafinil for the treatment of ADHD in adults is scarce.

**Objective:**

The present review aims to perform a systematized search of the available evidence on the possible therapeutic benefit of armodafinil treatment in adult patients with ADHD.

**Methods:**

A literature review using PubMed was conducted to compile and summarize the available clinical and scientific evidence on the possible use of armodafinil as a pharmacological treatment in adult patients with ADHD.

**Results:**

From the 86 articles reviewed, the available evidence showed that both acute and chronic treatment with armodafinil can improve wakefulness, memory, impulse control, and executive functions in adults with sleep disorders and other conditions. In addition, evidence of improvement in cognitive functions and mood alterations in other neuropsychiatric conditions was shown.

**Conclusion:**

Armodafinil could be useful for the treatment of ADHD in adults, according to the review of the literature from both pre-clinical and clinical studies.

## 1 INTRODUCTION

Attention deficit hyperactivity disorder (ADHD) is the most common neurodevelopmental condition during pediatric age. The symptomatology is observed from infancy, and it is estimated that up to 60% of patients continue to present symptoms and significant impairment during adulthood and childhood. This condition presents as a major clinical condition characterized by the presence of impairment of executive function, cognitive problems such as attention deficit, and a considerable increase in hyperactivity and impulsivity [1-3].

Prevalence estimates of ADHD vary according to age groups and diagnostic criteria. In the United States of America, it is estimated that about 9.4% of patients between 2 and 17 years of age are diagnosed with ADHD [4, 5], while in adulthood, there is an estimated 2.5% in the general population [6, 7], more frequently reported in men than in women in a ratio of 2:1 [8]. Previously, ADHD was thought to be a childhood condition; however, recent evidence indicates that significant symptoms continue to occur in adulthood, which are often difficult to distinguish from the symptoms of other neuropsychiatric conditions, making it more complex to adequately address and treat the condition.

Nowadays, ADHD symptomatology in adults is poorly studied and misunderstood by health professionals. Consequently, it is estimated that less than 20% of adults with ADHD are currently being diagnosed and treated by a psychiatric specialist, and those who seek care usually do so to treat some other comorbid condition, such as the presence of sleep disturbances or mood disorders [9]. Diagnosis and treatment are limited, and they are usually focused on modulating symptomatology related to the main comorbidities that patients present with, such as affective disorders, anxiety, personality, substance abuse, and eating disorders [9-11].

The first line of pharmacological treatment includes the use of psychostimulant drugs such as methylphenidate and amphetamine formulations such as lisdexamfetamine, which have shown high efficacy in patients of all ages [12, 13]. However, nearly 20 to 30% of patients report excessive side effects or do not respond to this line of treatment [14]. Non-psychostimulant drugs include atomoxetine, guanfacine, and clonidine, which are considered second-line treatments because they show a lower response and effect sizes and are usually reserved for patients who respond poorly or have significant side effects to first-line treatment [12, 13].

The lack of an effective, safe, not only in the short term but also in the long term, low addictive and long-lasting treatment highlights the current problem for the management of this condition and the reason why it is necessary to propose new therapeutic strategies that are safe and accessible to the population.

**Fig. (1):**
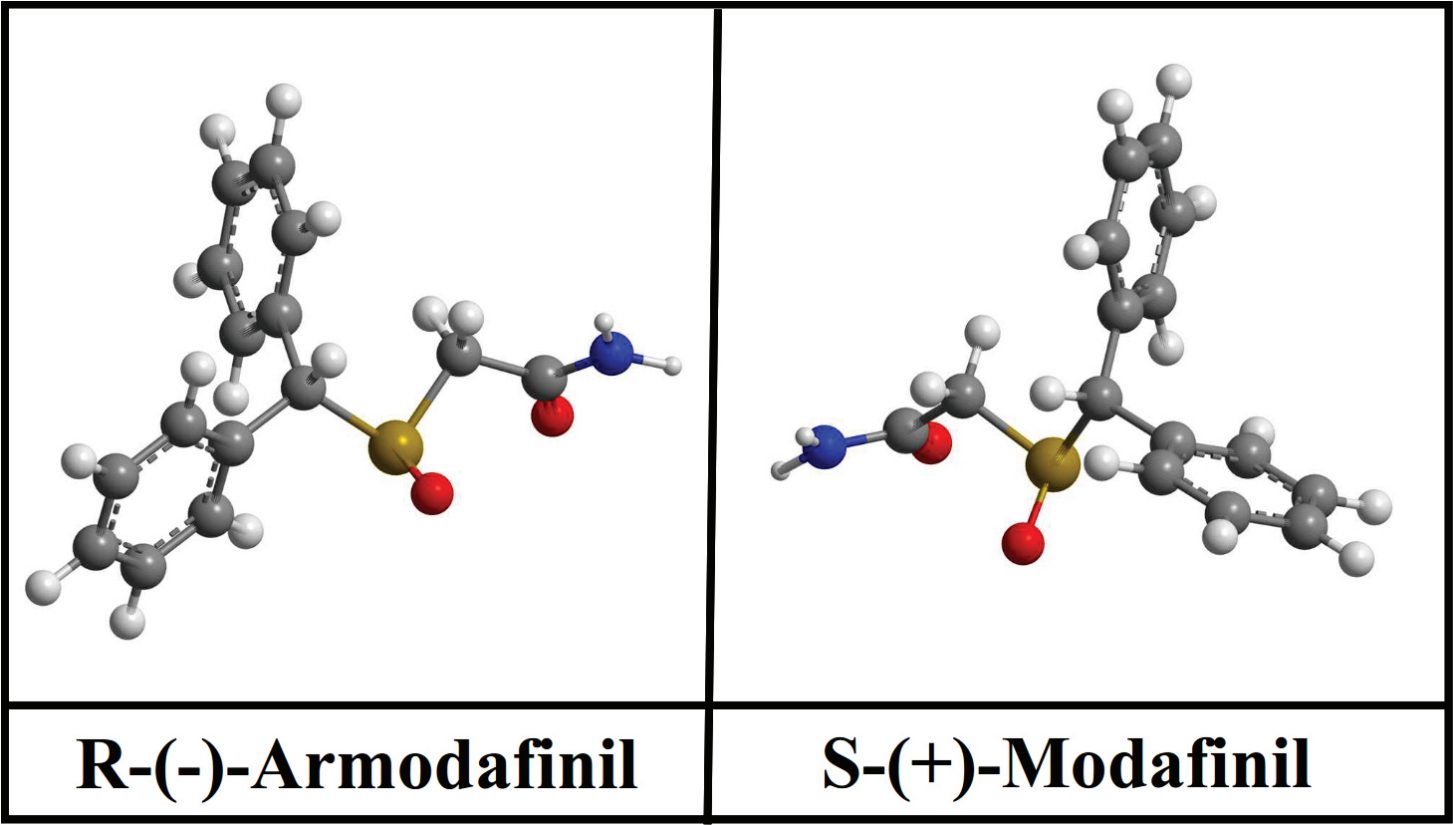
The R enantiomer (armodafinil) and S enantiomer (modafinil) chemical structure.

Armodafinil and its enantiomer S (modafinil) (Fig. **1**) have been described as psychostimulants with a different pharmacological profile, chemical structure, and mechanism of action than amphetamines, which have drawn attention in their use as an alternative treatment for the symptoms observed in ADHD, and this possibility could provide an answer to the current concern about the risk of dependence that has been reported with the abuse of psychostimulants [15-18]. There is evidence to suggest that these drugs improve function in several cognitive domains, including working memory and episodic memory, and other processes that depend on the prefrontal cortex and cognitive control in adults, as well as being well tolerated with a reduced rate of adverse events and risk of dependence [19]. This review aims to compile and summarize the available information on the efficacy of armodafinil treatment and the known neurobiological mechanisms involved in adult patients with ADHD.

## 2 METHODS OF SEARCH FOR THE INFORMATION

This review aims to compile and summarize the available evidence suggesting the potential use of armodafinil for the treatment of ADHD in adults. Clinical trial results and original studies in adult patients were included. The search for information was conducted using the National Library of Medicine, National Center for Biotechnology Information (NIH) PubMed. Logical functions and operators such as “or”, “and”, and “not” were included in the search using the following keywords: Attention-Deficit/Hyperactivity Disorder (ADHD), epidemiology of ADHD, sleep disorders and ADHD, attention, hyperactivity, psychostimulants, and ADHD, modafinil and ADHD, armodafinil and cognition, armodafinil and memory, armodafinil and sleep disorders. Articles written in languages other than English were excluded from the review. Selected studies that met the inclusion and exclusion criteria (86 articles) were analyzed and discussed in this review.

## 3 OVERVIEW OF ATTENTION DEFICIT/HYPER-ACTIVITY DISORDER (ADHD)

### 3.1 ADHD in Adults

ADHD is one of the most complex and prevalent neurodevelopmental conditions worldwide. It is a debilitating condition that generates important changes in behavioral patterns in adults [20]. Although the pathophysiological mechanisms underlying this disorder are not yet fully understood, available evidence suggests that there are several morphological alterations in the prefrontal, frontal, parietal, temporal, and entorhinal cortexes [21] and an imbalance of the noradrenergic and dopaminergic systems, especially in the frontal cortex [22].

Adult patients with ADHD show greater dysfunction in attention and higher executive functions, such as inhibitory control, emotional regulation, and cognitive flexibility, compared to the pediatric population, where hyperactivity and impulsivity generally predominate [23]. Therefore, the manifestations observed in adults with ADHD consist of a greater difficulty in maintaining focus and attention, leading to frequent forgetfulness and errors that cause frustration and significant discomfort, difficulties in planning, organizing, and executing tasks, an unpleasant physical restlessness that is reflected in the constant movement of the limbs, but also, greater difficulty in regulating emotions adequately [23, 24]. Likewise, a study published in 2006 indicates that adults with ADHD have a high rate of failure in school performance, work performance, and maintaining emotional bonds [25]. In support of the above, it has been observed that the clinical manifestations in adults with this condition have a direct impact on their functioning, as well as on the adequate development of interpersonal relationships, also hindering their social functioning [26]. On the other hand, major comorbid disorders such as substance abuse, especially those associated with earlier onset and more severe development, and various affective disorders appear to arise to some extent from a later onset as a consequence of primary ADHD, especially when the patient does not receive treatment promptly, which may contribute to misdiagnosis and delay in the patient's treatment and recovery [6, 26, 27].

ADHD is generally diagnosed using the Diagnostic and Statistical Manual of Mental Disorders (DSM-5) criteria [28]. The diagnostic criteria for ADHD focus on inattention and hyperactivity/impulsivity and can be classified into three different subtypes: inattentive (20-30% of cases), hyperactive-impulsive (15% of cases), and combined (50-75% of cases) [29]. However, currently, the symptom profile of adults with ADHD does not consider for its diagnosis the deficit of executive functions and emotional dysregulation [23].

Psychostimulant drugs such as methylphenidate and amphetamines are considered the first-line treatments, as they have a good tolerance profile [30, 31] and have shown to be effective in ADHD by inhibiting the reuptake of norepinephrine and dopamine [32]. These medications have an approximate duration of no more than 12 to 13 hours per dose, considering the extended-release formulations; thus, some patients require a combination of an extended-release and an immediate-release formulation to ensure that the effect lasts throughout the day [30, 33]. However, the most common side effects are appetite suppression, insomnia, dry mouth, and nausea. In addition, long-term treatment has been associated with alterations in growth trajectory, specifically in relation to height and weight [13]. For those patients who do not tolerate the first-line treatment of choice, non-stimulant therapy is used, including tricyclic antidepressants, α-agonists, clonidine, and guanfacine, among others [12, 34-36]. For this reason, stimulating agents are needed to treat those patients who do not respond satisfactorily to standard pharmacological treatment.

### 3.2 Circadian Rhythms and Sleep Disorders in Adult Patients with Attention-deficit/Hyperactivity Disorder (ADHD)

One of the main manifestations in adults with ADHD is sleep and circadian rhythm disturbances, which are associated with simple sleep-related movement disorders such as restless legs syndrome/periodic limb movements [37-39]. Sleep quality disturbances, *i.e*., insomnia and daytime sleepiness, are associated with inattention and hyperactivity in adults [40, 41]. This situation takes on special importance because untreated adults with ADHD have greater difficulty falling asleep and, therefore, a considerable increase in daytime attention problems [42]. The circadian system is hierarchically organized with a central pacemaker in the suprachiasmatic nucleus of the hypothalamus of the brain and is responsible for the generation of behavioral and physiological rhythms on a nearly 24-period basis and plays a key role in determining the rhythm of the sleep/wake cycle [43]. Previous studies indicate that in ADHD patients, circadian rhythm disturbance is closely related to dysfunction of melatonin and cortisol rhythms with subsequent decreased sleep duration and quality [44]. Melatonin and cortisol are key indicators in the sleep/wake cycle and are believed to be responsible for some of the altered behaviors characteristic of ADHD [45]. Likewise, it is known that deep circadian control and the alteration of dopaminergic neurotransmission are related; for this reason, the therapeutic approach to ADHD through the use of psychostimulants represents one of the first treatment alternatives [46]. Numerous studies have highlighted the importance of the use of psychostimulants for the treatment of ADHD in adults to decrease central symptomatology and behavioral problems.

### 3.3 Armodafinil

**Fig. (2):**
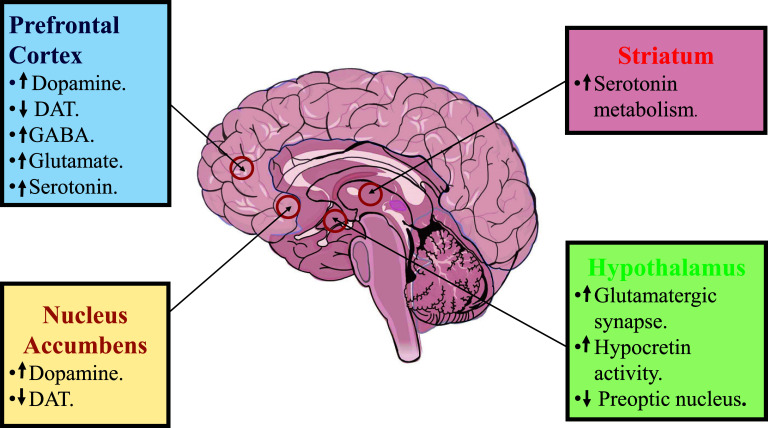
Mechanism of action of armodafinil: Prefrontal cortex, increase the concentration of dopamine (DA) through inhibition of dopamine transporter (DAT) and can promote GABAergic, glutamatergic and serotonergic activity. Nucleus accumbens, increase the concentration DA through inhibition of DAT. Striatum, increased serotonin metabolism. Hypothalamus, promotes glutamatergic synapse in hypocretin/orexin neurons and is capable of inhibiting activity of the preoptic nucleus.

The R enantiomer armodafinil is a non-amphetamine central nervous system stimulant that promotes wakefulness in adults and reaches highest plasma concentrations between 6 and 14 h after administration, with an associated longer duration of wakefulness-promoting activity in healthy adults [19]. It has a half-life of 10 to 14 h compared to 3-4 h induced by that of the S enantiomer (modafinil) [47]. The mechanism of action of armodafinil is not yet fully explained. However, its effects are attributed to an increase in the concentration of dopamine in the prefrontal cortex and nucleus accumbens as it binds to the dopamine transporter (DAT), improving executive functions such as attention, impulsivity, memory, and impulse control [48]. On the other hand, it has been suggested that armodafinil can promote glutamatergic synapses in hypocretin/orexin neurons in the lateral hypothalamus that regulate wakefulness effects [49]. In support of the above aspects, the available evidence focuses on the use of modafinil; consequently, this information indicates that the R enantiomer (armodafinil) may be a potential treatment for this condition (Fig. **2**).

#### 3.3.1 Modafinil as a Treatment for ADHD in Adults

Modafinil (2-[(diphenylmethyl) sulfinyl] acetamide) is an attention-promoting pharmacological agent that could be an effective and safe treatment option for ADHD. It is known to act on multiple areas of the attention and ascending arousal systems to increase frontal cortical activity [50]. The effects of commonly prescribed stimulant drugs are designed to mitigate ADHD symptoms during waking hours and have been shown to mitigate persistent problems related to planning, time awareness, and task prioritization.

Modafinil (S enantiomer) is a psychostimulant that exhibits potential as a cognitive enhancer in healthy adults and patients with ADHD. In healthy adult patients receiving a single dose of placebo or either 100 or 200 mg doses of modafinil, treated patients significantly improved their visual pattern recognition memory, spatial planning, and decreased impulsivity [51]. With the same aim, a double-blind, randomized, placebo-controlled crossover study using a single 200 mg dose of modafinil produced a significant pattern of improvement in short-term memory and visual memory ability in adult patients diagnosed with ADHD [14]. Another randomized, double-blind, placebo-controlled study reported that treatment with modafinil (206.8 mg/day) improved ADHD symptoms with results similar to those obtained with dextroamphetamine treatment (21.8 mg/day) [52]. In addition, modafinil has been reported to be well tolerated in children and adolescents and significantly improved ADHD symptomatology [53, 54]. In general, modafinil is well tolerated, and the adverse reactions reported were insomnia, headache, and decreased appetite [55, 56]. This evidence supports the idea that armodafinil may be beneficial in the treatment of ADHD; however, it highlights the importance of conducting preclinical and clinical trials to support it as a therapeutic alternative.

#### 3.3.2 Armodafinil as a Possible Treatment for Adult ADHD

In addition to the core symptoms that are commonly present in adults diagnosed with ADHD, such as attention difficulties and hyperactivity, there are those related to sleep disturbances and circadian rhythm. The changes caused by circadian rhythms generate complications associated with cognitive and executive function [57]. Previous reports indicate that armodafinil is safe and effective for the treatment of excessive sleepiness induced by shift work disorders or narcolepsy [58, 59]. With the same objective, several studies in healthy adult men indicated that treatment with armodafinil (250 mg) improved alertness in sleep-deprived patients compared to the group of patients who received only placebo treatment [60, 61]. Rosenberg *et al.* reported that armodafinil was generally well tolerated at a dosage of 150 mg/day and increases wakefulness after eastbound travel through 6 time zones in patients with a history of jet lag symptoms [62].

In addition, reports indicate that prolonged treatment with armodafinil (150 mg) for 12 weeks is well tolerated and is associated with significant improvement in memory, attention, and fatigue in patients aged 18 to 65 years with narcolepsy [63]. On the other hand, it has been observed that prolonged treatment with armodafinil (150 mg/day) for 12 weeks significantly improved long-term memory, patient-estimated wakefulness, and reduced fatigue compared to placebo in patients with obstructive sleep apnea/hypopnea syndrome [47], while in patients with obstructive sleep apnea, it significantly improved wakefulness, long-term memory and patients' ability to participate in activities of daily living [64]. Another study showed that treatment with armodafinil (150 mg/day) reduced chronic fatigue in patients undergoing radiotherapy [65]. A double-blind, placebo-controlled, crossover study demonstrated that treatment with armodafinil (250 mg) significantly improved verbal memory and learning in patients with multiple sclerosis [66].

Another study indicated that armodafinil (150 mg) significantly improved depressive symptoms compared to placebo in patients with a major depressive episode associated with bipolar I disorder [67]. Safety data for treatment with armodafinil (150 mg) showed that it was well tolerated, and the most common adverse reactions that occurred were headache, insomnia, nausea, and diarrhea [62, 67, 68] (Table **1**) and several reports indicate that treatment with armodafinil shows a pharmacological profile similar to that observed with the use of conventional psychostimulants used as first-line treatment for ADHD, as it has proven to be a cognitive enhancer in diverse pathologies characterized by inducing cognitive impairment about executive function, fatigue and sleep disorders [70-75] (Table **2**).

## 4 ARMODAFINIL IN EXPERIMENTAL ANIMAL MODELS

### 4.1 Armodafinil as a Promoter of Wakefulness and Cognitive Enhancement in Animals

Clinical studies have reported that armodafinil is a useful neurostimulant for improving cognitive impairment and sleep disorders [76]. Wisor *et al.* reported that the intraperitoneal administration of 100 mg/kg of armodafinil to rats significantly increased the time of wakefulness and locomotor activity evaluated by electroencephalographic signals when compared with those of the control group that did not receive the treatment. It was also observed that this effect was similar to that obtained with D-methamphetamine administered at a dose of 1 mg/kg, ip, a classic neurostimulant usually used for treating excessive sleepiness [77]. This effect is significant because sleep deprivation decreases neurogenesis and reduces the size of the hippocampus, leading to cognitive impairment, mainly affecting learning and memory [78]. On the other hand, a more up-to-date study by Zhu *et al*. observed that intranasal administration of armodafinil (30 mg/kg) in sleep-deprived rats presented a shorter escape latency and longer transfer times in the Morris water maze, as well as an increase in the central distance and vertical position of the animals in the open field test and increased expression of brain-derived neurotrophic factor (BDNF) in the hippocampus when compared to sleep-deprived rats that did not receive armodafinil as treatment; these data demonstrate that treatment with armodafinil could improve cognition and wakefulness in these animals [79].

While, in a study by Fiocchi *et al.* reported that treatment with armodafinil (100 mg/kg, i.p.) increases wakefulness in rats and is associated with an increased number of neurons labeled with the transcription factor c-Fos, a marker of functional activity, both in the striatum and the anterior cingulate cortex, regions closely related to the activation of the dopaminergic system during waking states. This finding is relevant, as it has been reported that neurons of the ventrolateral preoptic nucleus (VLPO) of rats increase c-Fos activity after sleep, promoting GABAergic innervation of the main monoamine excitatory systems so that VLPO lesions are related to the onset of insomnia in animals [81]. Likewise, Vetrivelan *et al.* concluded that armodafinil administered intraperitoneally at a dose of 200 mg/kg promotes long-lasting wakefulness states in rats with VLPO lesions [82].

Due to a high content of polyunsaturated fatty acids, brain tissue is very sensitive to neurodegeneration processes after oxidative stress. The ability of cells to counteract cellular stress is regulated by protective genes known as vitagenes, capable of activating specific pro-survival cellular pathways [83]. During the last decades, special interest has been shown in Nuclear Factor Erythroid 2 (NRF-2) because it constitutes a key transcription factor for the regulation of redox homeostasis as well as in the activation of multiple mechanisms that protect against neuroinflammation and promotes neuronal plasticity [84]. This adaptive process is regulated by mechanisms like the hormetic effect in which a dose response or concentration may be induced for a mild stress response and cause a positive adaptive response, while higher concentrations evoke dysfunctional or toxic effects [85].

Armodafinil has been observed to have a neuroprotective effect by preventing memory loss after administration of scopolamine (3 mg/kg) as a model of Alzheimer's disease. That was observed by Katta *et al.* in a model of Alzheimer's disease induced with scopolamine (3 mg/kg) showed that treatment with armodafinil at an oral administration dose of 30 mg/kg once a day for 15 days decreased cognitive impairment, and increased spatial memory compared to the group of animals that did not receive the treatment. In addition, they reported that the treatment with armodafinil improves oxidative stress to decreased lipid peroxidation and increased glutamate concentration, additionally increased acetylcholinesterase levels, and effects associated with an improvement in learning and memory [86]. All those findings support the hypothesis relating oxidative stress and the pathophysiology of neurodegenerative and neurodevelopmental disorders such as ADHD.

## CONCLUSION

The main findings of this review indicate that impairments in the dopaminergic system and circadian cycles play a central role in the pathophysiology of ADHD; for this reason, the use of psychostimulant drugs remains one of the main therapies for the management of symptomatology. However, amphetamine drugs and methylphenidate offer temporary relief of symptomatology and represent a risk of abuse for the population. Currently, there is increased interest in the use of drugs to help mitigate ADHD symptoms that are safe, effective, and well-accepted by patients. Following this review, we found numerous studies suggesting that armodafinil may be a good candidate for long-term treatment in adults with ADHD, as it has been shown to have several therapeutic benefits on cognition and alertness in several pathologies that share similarities with ADHD. However, the use of this psychostimulant to alleviate symptoms such as fatigue, drowsiness and inactivity is inconclusive. The evidence collected suggests that armodafinil is well tolerated at doses ranging from 50 to 250 mg, with mostly adverse events such as insomnia, headache, and decreased appetite. Armodafinil may have advantages over current therapies, including amphetamines and methylphenidate, because it has a neurobiological profile capable of modulating alertness and wakefulness. Armodafinil could potentially be an emerging treatment option for ADHD; however, the lack of comparative and long-term clinical studies with current treatments demonstrating its therapeutic effectiveness hinders its use in patients with this condition. The information contained in this review article highlights the importance of the correct identification of the symptomatology to generate new long-term therapeutic strategies; likewise, the management of sleep problems in ADHD patients with armodafinil could represent an alternative that allows adult patients to substantially improve their quality of life.

## Figures and Tables

**Table 1:** Safety and effectiveness of the different psychostimulants.

**Treatment**	**References**	**Study Type**	**Evaluation Method**	**Main Side Effects**	**Main Finding**
Armodafinil	[68]	Multicenter, prospective, randomized, double-blind, placebo-controlled	CANTAB Vital parameters (heart rate, systolic and diastolic blood pressure)	Headache (19%), nasopharyngitis (14%), and diarrhea (10%)	Armodafinil was generally well tolerated but with 200 mg/day for 2 weeks did not improve work memory in 21 patients with obstructive sleep apnea (OSA) and excessive sleepiness
	[62]	Double-blind, randomized, parallel-group, multicenter study	Multiple Sleep Latency Test, CGI, Karolinska Sleepiness Scale and Nocturnal Polysomnography	Headache (27%), nausea (13%), diarrhea (5%), circadian rhythm sleep disorder (5%), and palpitations (5%)	Armodafinil was generally well tolerated and at a dosage of 150 mg/day for three days increases wakefulness in 427 patients with a history of symptoms of jet lag
Modafinil	[55]	A Double-Blind, Randomized, Placebo-Controlled Study	CANTAB	Headache, increased anxiety, drowsiness, sleep disturbance	Modafinil was well tolerated and report improvements on test of episodic memory and working memory but with a single dose of 200 mg did not improve the attention and the executive functions in 30 patients with remitted depression
	[14]	Double-blind, randomized, placebo-controlled crossover study	CANTAB	Patients reported feeling of excited	Modafinil was well tolerated and improvements on tests of short-term memory span, visual memory, spatial planning, and stop-signal motor inhibition with a single dose of 200 mg in 20 adults with ADHD
Methylphenidate (MPH)	[69]	Pilot study randomized placebo-controlled	Reverse Digit Span Test Trail making test Bochum matrices-advanced test	Patients reported sleep-onset and sleep maintenance insomnia, headache and restlessness	MPH Improved in fatigue and declarative memory with two sessions separated by approximately one week of 20 mg immediate-release methylphenidate in 48 healthy volunteers
	[70]	Large, prospective, randomized multi-center clinical trial	Vital parameters (heart rate, systolic and diastolic blood pressure, body weight) EKG	Decreased appetite (22%) dry mouth (15%), palpitations (13%), gastrointestinal infection (11%), agitation (11%), restlessness (10%), hyperhidrosis, tachycardia, weight decrease (6.3%)	MPH was safe and well-tolerated with a dose of 10mg for long term (52 weeks) treatment in 205 adults with ADHD
Lisdexamfetamine (LDX)	[71]	Open label multicenter, Double-blind placebo-controlled trial	Vital parameters (heart rate, systolic and diastolic blood pressure) EKG ADHD-RS CGI	87.7% presented any TAESs: Upper respiratory tract infection 21.8%, insomnia 19.5%, headache 17.2%, dry mouth 16.6%, decrease appetitive 14.3%, irritability 11.2%, anxiety 8.3%	LDX was generally well tolerated and efficacious with a dose of 30 mg to 70 mg/day for long term (12 months) treatment in 349 (191 completed the trial) adults with ADHD
	[72]	Open label multicenter, randomized, double-blind, placebo-controlled	Vital parameters (heart rate, systolic and diastolic blood pressure, height and weight) EKG ADHD-RS, CGI, YQOL-R	86.7% presented (TAESs) 10% presented common TEAEs: decreased appetite (21.1%), headache (20.8%), insomnia (12.1%), and dizziness (5.3%) 4.9% experienced 18 severe TEAEs included dizziness, headache, migraine, aggression, agitation, dermatitis contact	LDX was generally safety and effectiveness with a dose of 30 mg, 50 mg and 70 mg/day For long term (12 months) treatment in 198 (119 completed the trial) adolescents with ADHD

**Abbreviations:** CANTAB = Cambridge neuropsychological test automated battery, CGI = The clinical global impression, EKG = electrocardiogram, ADHD-RS = ADHD Rating Scale-IV, YQOL-R = The Youth Quality of Life-Research Version, TAESs = treatment emergent adverse events.

**Table 2:** Effects of treatment with armodafinil on behaviors associated with ADHD.

**Study Type**	**Treatment**	**Pathology**	**Effect**	**References**
Double-blind, placebo-controlled, crossover study	Armodafinil unique dose (250 mg)	Multiple Sclerosis	Improves delayed verbal recall in 17 patients with Multiple Sclerosis	[66]
Randomized, double-blind, placebo-controlled trial	Armodafinil 6 weeks (250 mg/day)	Schizophrenia or schizoaffective disorder	Improved anhedonia-asociality in 60 patients with schizophrenia or schizoaffective disorder	[73]
Pilot trial	Armodafinil 12 weeks (125-250 mg/day)	Dementia with Lewy bodies	Improvements in hypersomnia and wakefulness in 20 patients with Dementia with Lewy Bodies	[74]
Randomized, placebo-controlled, double-blind trial followed by open-label extension.	Armodafinil 12 weeks (50-250 mg/day)	Mild to moderate closed traumatic brain injury	Significantly improved sleep latency in 88 patients with excessive sleepiness associated with mild to moderate traumatic brain injury	[75]

## LIST OF ABBREVIATIONS

ADHD: Attention Deficit Hyperactivity Disorder
BDNF: Brain-derived Neurotrophic Factor
DAT: Dopamine Transporter
NRF-2: Nuclear Factor Erythroid 2
VLPO: Ventrolateral Preoptic Nucleus
