# Corrigendum to: Armodafinil as a Potential Pharmacological Treatment for Attention Deficit Hyperactivity Disorder in Adults: A Review

**DOI:** 10.2174/1570159X23999250210110914

**Published:** 2025-02-10

**Authors:** Reyna Lamas-Aguilar, Araceli Diaz-Ruiz, Luz Navarro, Raúl Miranda-Ojeda, María de los Ángeles Martínez-Cárdenas, Alfonso Mata-Bermudez, Camilo Rios

**Affiliations:** 1 Departamento de Neuroquímica, Instituto Nacional de Neurología y Neurocirugía Manuel Velasco Suárez, Ciudad de México, México;; 2 Departamento de Fisiología, Facultad de Medicina, Universidad Nacional Autónoma de México, Ciudad de México, México;; 3 The Mind Project, Office for Equity, Diversity, Inclusion, and Belonging, Harvard University, Smith Campus Center, Cambridge, Massachusetts, USA;; 4 Faculty of Medicine, Autonomous University of Mexico State, Toluca de Lerdo, Estado de Mexico, Mexico;; 5 Departamento de Atención a la Salud, Universidad Autónoma Metropolitana Unidad Xochimilco, Ciudad de México, México;; 6 Dirección de Investigación, Instituto Nacional de Rehabilitación Luis Guillermo Ibarra Ibarra., Ciudad de México, México

The author has identified an error in the department of the authors in the article titled, Armodafinil as a Potential Pharmacological Treatment for Attention Deficit Hyperactivity Disorder in Adults: A Review”, published in Current Neuropharmacology, 2024, *22*(11), 1899-1908 [1].

Details of the error and a correction are provided here.

## ORIGINAL

**RAÚL MIRANDA-OJEDA^3,4^**

^3^The Mind Project, Office for Equity, Diversity, Inclusion, and Belonging, Harvard University, Smith Campus Center, Cambridge, Massachusetts, USA;

^4^Faculty of Medicine, Autonomous University of Mexico State, Toluca de Lerdo, Estado de Mexico, Mexico.

## CORRECTED

**RAÚL MIRANDA-OJEDA^3,4^**

^3^Department of Physical Medicine & Rehabilitation, Harvard Medical School, Boston, Massachusetts, United States of America;

^4^Faculty of Medicine, Autonomous University of the State of Mexico, Av Paseo Tollocan, C. Jesu´s Carranza, Moderna de La Cruz, 50180 Toluca de Lerdo, Mexico.

Publisher regret the error and apologize to readers.

The original article can be found online at: https://www.benthamscience.com/article/138216.
